# Serotonin and the regulation of mammalian energy balance

**DOI:** 10.3389/fnins.2013.00036

**Published:** 2013-03-27

**Authors:** Michael H. Donovan, Laurence H. Tecott

**Affiliations:** Department of Psychiatry, University of California San FranciscoCA, USA

**Keywords:** serotonin, energy balance, feeding behavior, leptin, ghrelin, orexin, insulin, hypothalamus

## Abstract

Maintenance of energy balance requires regulation of the amount and timing of food intake. Decades of experiments utilizing pharmacological and later genetic manipulations have demonstrated the importance of serotonin signaling in this regulation. Much progress has been made in recent years in understanding how central nervous system (CNS) serotonin systems acting through a diverse array of serotonin receptors impact feeding behavior and metabolism. Particular attention has been paid to mechanisms through which serotonin impacts energy balance pathways within the hypothalamus. How upstream factors relevant to energy balance regulate the release of hypothalamic serotonin is less clear, but work addressing this issue is underway. Generally, investigation into the central serotonergic regulation of energy balance has had a predominantly “hypothalamocentric” focus, yet non-hypothalamic structures that have been implicated in energy balance regulation also receive serotonergic innervation and express multiple subtypes of serotonin receptors. Moreover, there is a growing appreciation of the diverse mechanisms through which peripheral serotonin impacts energy balance regulation. Clearly, the serotonergic regulation of energy balance is a field characterized by both rapid advances and by an extensive and diverse set of central and peripheral mechanisms yet to be delineated.

## Central serotonin and energy balance

The monoamine signaling molecule 5-hydroxytryptamine (5-HT, serotonin) is utilized by diverse invertebrate and vertebrate species for the regulation of many of the behavioral and physiological processes through which energy balance is maintained (Horvitz et al., 1982; Orchard, 2006; Tecott, 2007). With regard to feeding, the predominant global effect of central nervous system (CNS) serotonin signaling is the suppression of food intake. In mammals, CNS serotonin is synthesized exclusively in discrete collections of brainstem neurons known as the raphe nuclei. Serotonergic raphe neurons project widely and extensively, delivering serotonin throughout the CNS. The caudal raphe nuclei send predominantly descending projections to the brainstem and spinal cord, including to areas important for energy balance such as the nucleus of the solitary tract (NTS) and the parabrachial nuclei (PBN; Lam and Heisler, 2007). The rostral raphe nuclei, including the median and dorsal raphe, send predominantly ascending projections throughout the forebrain, including to the cortex, amygdala, striatum, hippocampus, and the various nuclei of the hypothalamus. It has long been established that most areas of the hypothalamus receive inputs from both the median and dorsal raphe (Sawchenko et al., 1983; Willoughby and Blessing, 1987; Petrov et al., 1992).

Either specific lesions of raphe nuclei or acute inhibition of these neurons by raphe injection of the gamma-aminobutyric acid receptor A (GABA-A) agonist muscimol resulted in hyperphagia and obesity (Geyer et al., 1976; Klitenick and Wirtshafter, 1988). Furthermore pharmacological perturbation of serotonin synthesis by intracerebroventricular (ICV) injection of either the serotonergic neurotoxin 5,7-dihydroxytriptamine (5,7-DHT) or the tryptophan hydroxylase (TPH) inhibitor p-chlorophenylalanine (PCPA) have also been found to produce hyperphagia (Breisch et al., 1976; Saller and Stricker, 1976).

Conversely, central injections of serotonin or its precursor 5-hydroxytryptophan (5HTP) caused hypophagia (Blundell and Latham, 1979; Fletcher and Burton, 1986; Simansky, 1996; Yamada et al., 2006) as well as increased metabolic rate (Rothwell and Stock, 1987; Le Feuvre et al., 1991). Fenfluramine, a drug that increases synaptic serotonin concentrations by inducing vesicular release and inhibiting reuptake, also produced hypophagia, reducing meal size, and increasing inter-meal intervals in rats (Blundell et al., 1975; Grinker et al., 1980), effects which are mediated by central serotonin (Fletcher and Burton, 1986). Additionally, metabolic rate was also increased by fenfluramine administration (Rothwell and Stock, 1987; Le Feuvre et al., 1991). Moreover, the biologically active metabolite of fenfluramine, norfenfluramine, acts directly as an agonist at several serotonin receptor subtypes (Curzon et al., 1997). Fenfluramine suppressed eating and facilitated body weight loss in humans (Rogers and Blundell, 1979; McGuirk et al., 1991), and was combined with phentermine, a catecholamine/serotonin releasing agent in “Fen-Phen,” a widely prescribed weight loss treatment. “Fen-Phen” was removed from the market in 1997 due to its promotion of cardiac valvulopathies, now attributed to action at 5HT2B receptors expressed on cardiac valves (Fitzgerald et al., 2000; Rothman et al., 2000).

While a preponderance of evidence points to the inhibition of feeding by serotonin, there are a few recent results that are difficult to square with such a simple model. These include several genetic perturbations of the serotonin system as well as pharmacological manipulation of several different serotonin receptors (5HTRs; discussed in a subsequent section). Interpretation of adult phenotypes in the genetic models is complicated by the fact that serotonin has significant roles in development (Alenina et al., 2009). A recent study found that mice lacking expression of the CNS serotonin synthetic enzyme TPH2 displayed decreased food consumption and body weight (Yadav et al., 2009). However, other studies of independently generated TPH2 knockout mice reported mixed results. Some have also reported modest decreases in body weight or body fat (Gutknecht et al., 2008; Savelieva et al., 2008), while others have found no differences (Alenina et al., 2009; Liu et al., 2011). Two additional studies utilizing different genetic manipulations that result in depleted serotonin also reported no changes in body weight (Hendricks et al., 2003; Narboux-Neme et al., 2011).

A second line of evidence at odds with the notion that experimentally enhancing serotonin inhibits feeding involves genetic manipulation of the serotonin reuptake transporter (SERT or 5-HTT). As expected, the absence of serotonin reuptake into presynaptic terminals enhanced synaptic concentrations of serotonin, and SERT null mice exhibited elevated extracellular serotonin levels (Mathews et al., 2004). Interestingly, though, mice lacking SERT had an adult-onset obesity phenotype (Warden et al., 2005; Murphy and Lesch, 2008). While these mice were not hyperphagic, they were hypoactive, with reduced home-cage locomotor activity (Holmes et al., 2002). The phenotype was reversed for transgenic mice that overexpress SERT at 2–3 fold normal levels, with a consequent decrease in brain extracellular serotonin (Jennings et al., 2006). These mice had reduced body weight and also no alteration in feeding (Pringle et al., 2008).

It seems likely that some of the discrepancies between pharmacological and genetic manipulations of serotonin systems reflect differences between acute effects on one hand and chronic or possibly developmental effects on the other. It is worth noting that many of the transgenic mice with dramatic reductions in serotonin synthesis exhibited relatively minor behavioral abnormalities during adulthood (Savelieva et al., 2008). This rather surprising result raises the possibility that there may be some significant compensatory mechanisms at work in serotonergic target neurons or downstream feeding circuits.

## The 5HT2C receptor

Serotonin exerts its effects through actions involving at least 18 different 5HTRs, all but one of which are G-protein coupled receptors (Marston et al., 2011). Within the hypothalamus, no serotonin receptor is more highly expressed than the 5HT2CR (Yadav et al., 2009). A role for 5HT2CR-mediated signaling in energy balance was first indicated by studies showing that a non-specific serotonin receptor agonist with high affinity at 5HT2CRs, m-chlorophenylpiperazine (mCPP) decreased feeding and that this effect was blocked by non-selective antagonists with high affinities for the 5HT2CR (Kennett and Curzon, 1988, 1991; Kitchener and Dourish, 1994). The anorectic action of 5HT2CR was subsequently confirmed through studies of 5HT2CR knockout mice that lacked functional 5HT2CRs. These animals displayed chronic hyperphagia leading to late onset obesity (Tecott et al., 1995; Nonogaki et al., 1998). Moreover, they exhibited reduced sensitivity to the anorectic effects of dexfenfluramine (Vickers et al., 1999).

The natural course of obesity development in 5HT2CR knockout mice is reminiscent of common forms of human obesity. These animals display chronic hyperphagia (Tecott et al., 1995; Nonogaki et al., 1998), however, they do not develop obesity until 5–6 months of age (Nonogaki et al., 1998), possibly because they also exhibit enhanced home cage locomotor activity. Although the hyperlocomotor phenotype persists in older 5HT2CR knockout mice, their development of late onset obesity may be attributable to progressive increases in the energy efficiency of locomotor activity with increasing age (Nonogaki et al., 2003). These animals also display enhanced sensitivity to the obesigenic effects of high-fat feeding, including the development of type 2 diabetes mellitus (Nonogaki et al., 1998). Moreover, detailed analysis of behavior patterns in 5HT2CR null mice revealed that their hyperphagia occurred during a portion of the light cycle during which mice typically exhibit inactivity (Goulding et al., 2008). During this period, mutants were observed to exhibit multiple brief periods of activity characterized by visits to the feeder. This pattern of multiple brief feeding episodes during the inactive portion of the circadian cycle is reminiscent of the human night-eating syndrome, a condition responsive to serotonin reuptake blockade (O'Reardon et al., 2006). Altogether, a number of features of the obesity syndrome in 5HT2CR knockout mice resemble common forms of human obesity: (1) chronic hyperphagia, (2) lack of primary perturbations of metabolic rate, (3) lack of primary perturbations of the regulation of hormones implicated in energy balance, (4) late-onset, (5) exacerbation by high levels of dietary fat, and (6) a circadian pattern resembling the human night-eating syndrome. The multiple lines of evidence indicating a prominent role of 5HT2CRs in the serotonergic suppression of feeding led this receptor to be targeted for the development of agonists for obesity treatment. Recently, the 5HT2CR-specific agonist lorcaserin became the first FDA approved anti-obesity drug in 13 years (Lam et al., 2008; Thomsen et al., 2008; Hurren and Berlie, 2011).

Another intriguing feature of 5HT2CR biology relates to epigenetic modification of the *Htr2c* gene product; it is one of a limited number of identified gene products known to be subject to RNA editing (Rula and Emeson, 2007). Editing occurs at five adenosine bases (editing sites A–E) within a 13 base span of exon V of the *Htr2c* gene, in a protein-coding region corresponding to the second intracellular loop of the receptor (Burns et al., 1997). This edited region is regarded as critical for signal transduction through G-protein mediated intracellular pathways (Werry et al., 2008). The edited region is also located close to a splice donor site, and alternative splicing at this site produces a truncated non-functional gene product lacking part of exon V (*Htr2c-tr*) (Canton et al., 1996; Xie et al., 1996). There are 32 possible editing combinations arising from these 5 sites, resulting in 24 possible amino acid sequences, ranging from the non-edited (INI) to the fully edited (VGV) form. 5HT2CR RNA editing has functional consequences: generally, an inverse relationship exists between the extent of editing and 5HT2CR constitutive activity (Burns et al., 1997; Herrick-Davis et al., 1999; Niswender et al., 1999).

Several lines of evidence implicate perturbations of 5HT2CR RNA editing in the marked hyperphagia associated with Prader–Willi Syndrome (PWS), a developmental disorder resulting from loss of paternal gene expression on chromosome 15q11–13. The disorder is characterized by cognitive impairment, short stature, and hyperphagia often leading to morbid obesity (Nicholls and Knepper, 2001; Goldstone, 2004). Intriguingly, the deleted region of 15q11–13 encodes multiple copies of SNORD115 (also known as H/MBII-52), a small nucleolar RNA (snoRNA) containing an 18 nucleotide anti-sense box complementary to the edited region of 5HT2CR pre-mRNA. Moreover, SNORD115 can modulate both 5HT2CR editing and alternative splicing (de los Santos et al., 2000; Cavaille et al., 2001). Consistent with a potential role for perturbation of 5HT2CR editing in the pathophysiology of PWS, brain samples from PWS patients exhibited altered editing patterns (Kishore and Stamm, 2006). Moreover, a line of mice bearing a deletion of *Snord115* exhibited altered 5HT2CR editing patterns, perturbations of feeding and additional behaviors relevant to PWS (Doe et al., 2009). Another line of mice solely expressing the fully edited isoform of 5HT2CR also exhibited disorders of feeding and additional behaviors relevant to PWS (Morabito et al., 2010). Finally, leptin deficient *ob/ob* mice have been recently found to exhibit perturbations of 5HT2CR editing within the hypothalamus, raising the possibility that the regulation of 5HT2CR editing may be sensitive to energy balance perturbations (Schellekens et al., 2012). It will be of great interest to determine in future studies the extent to which 5HT2CR RNA editing processes are sensitive to energy status.

## Serotonin and the hypothalamus

While many areas of the brain have demonstrated roles in transmitting and integrating energy balance signals, the hypothalamus is pivotal. This small region of the limbic system also has a central role in mediating stress responses, regulating body temperature, thirst and sleep, and establishing circadian rhythms. It is bordered by the third ventricle and the highly vascularized median eminence, an area with a porous blood-brain barrier. This makes it ideally positioned to sense and respond to a myriad of circulating hormones and nutrients (Rodriguez et al., 2010). The hypothalamus also receives major innervation from the NTS, a brainstem structure that relays signals transmitted via the vagus nerve from the gastrointestinal tract and other visceral organs (Sawchenko et al., 1985; Cunningham and Sawchenko, 1988). Additionally, various areas of the hypothalamus receive a wide range of projections from other areas of the brain involved in energy balance, including other brainstem nuclei, olfactory cortex, and reward-related areas such as the ventral striatum (Gao and Horvath, 2007).

Early experiments in rats examining the effects of discrete lesions of hypothalamic subregions reported an interesting functional division between the medial and lateral hypothalamus: lesions of medial nuclei including the paraventricular (PVH), dorsomedial (DMH), and ventromedial (VMH) nuclei produced hyperphagia and obesity while lesions within the lateral hypothalamic area (LHA) produced hypophagia (Hetherington and Ranson, 1940; Brobeck et al., 1943; Anand and Brobeck, 1951). This led to the hypothesis that the hypothalamus consisted of two centers with opposing influences on behavior: a satiety center, located medially and a feeding center, located laterally. Experiments utilizing the molecular and genetic tools of subsequent decades have demonstrated that hypothalamic physiology is somewhat more complex, integrating diverse signals reflecting short- and long-term energy stores, ingestion and digestion, circadian patterns, and environmental cues to dictate when and how much feeding occurs as well as to modulate activity and metabolic rate.

One population of neurons that seems to have an especially significant role in mediating energy balance signals is located in the arcuate nucleus and expresses proopiomelanocortin (POMC). The arcuate nucleus is located at the highly vascularized ventromedial aspect of the hypothalamus, immediately adjacent to the median eminence, allowing access to a wide range of circulating factors (Cone et al., 2001; Rodriguez et al., 2010). POMC neurons within the arcuate play an important role in sensing and integrating these peripheral factors (Williams et al., 2011). The POMC protein is enzymatically cleaved into several secreted peptides, including alpha melanocyte-stimulating hormone (αMSH) (Cone, 2005). αMSH is released onto downstream neurons in the DMH, VMH, PVH, and LHA, where it serves as an agonist for melanocortin receptors (MCR), in particular MC3R and MC4R (Adan et al., 1994). Genetic disruption of either POMC or MC4R has been shown to produce a dramatic increases in feeding and weight gain, indicating that a primary function of this melanocortin system is anorectic (Huszar et al., 1997; Krude et al., 1998).

POMC neurons of the arcuate nucleus express 5HT2CRs (Heisler et al., 2003; Lam et al., 2008) and both 5HT2CR agonists and d-fenfluramine stimulate POMC neuronal activity (Heisler et al., 2002). Furthermore, the hypophagia and weight loss produced by these serotonergic agents are suppressed by either genetic or pharmacological inactivation of melanocortin circuits (Heisler et al., 2002, 2003; Nonogaki and Kaji, 2010). The requirement for both an intact melanocortin circuit and functional 5HT2C receptors (Vickers et al., 1999) for the anorectic effectiveness of fenfluramine led to the hypothesis that 5HT2CR on POMC neurons may play a particularly important role in mediating the energy balance effects of serotonin. This hypothesis has been largely confirmed in recent studies (Xu et al., 2008, 2010a). These studies utilized a transgenic strategy whereby 5HT2CRs were expressed specifically on POMC neurons in an otherwise 5HT2CR null background. 5HT2CR expression solely on POMC neurons ameliorated energy balance phenotypes of 5HT2CR null mice including: hyperphagia, sensitivity to diet-induced obesity, locomotor hyperactivity, insulin resistance, and insensitivity to the anorectic effects of serotonin agonists (Xu et al., 2008, 2010a,b). Given the presence of 5HT2CRs on other neuronal populations involved in energy balance, it is somewhat surprising that receptors solely on POMC neurons were sufficient for restoration of normal energy balance. While this strategy demonstrates that POMC 5HT2CRs were sufficient to ameliorate phenotypes resulting from *Htr2c* gene inactivation, it will also be of interest to determine whether they are necessary for intact energy balance regulation in an otherwise normal brain. That is, whether inactivation of 5HT2CRs specifically on POMC neurons will produce energy balance phenotypes similar to those observed in the mice globally lacking 5HT2CRs.

In addition to POMC cells, a second type of arcuate nucleus neuron has been implicated in serotonergic control of energy balance. These neurons produce agouti-related peptide (AgRP), an endogenous antagonist of MC3R and MC4R (Nijenhuis et al., 2001; Chai et al., 2003), as well as gamma-aminobutyric acid GABA and neuropeptide Y (NPY), both of which inhibit POMC neurons and downstream melanocortin target neurons (Cowley et al., 2001). The net result of both of these effects is orexigenic behavior. Central administration of NPY or overexpression of AgRP increased food consumption leading to obesity (Stanley and Leibowitz, 1985; Ollmann et al., 1997). Interestingly genetic AgRP and NPY nulls, as well as double nulls, did not produce the expected hypophagia phenotype (Erickson et al., 1996; Qian et al., 2002). However, experiments utilizing complex genetic tools to inducibly ablate AgRP neurons, revealed that ablation during adulthood, but not neonatally, produced extreme hypophagia, rapidly leading to starvation (Gropp et al., 2005; Luquet et al., 2005). These results indicated that significant compensation likely occurred in the AgRP null mice, highlighting the redundancy in these systems and therefore the importance of robust pro-feeding circuits to an animal's survival.

5HT1BRs are expressed in arcuate nucleus AgRP neurons and have been implicated in their regulation. When expressed on serotonergic neurons, the G_i_-coupled 5HT1BR acts as an autoreceptor, inhibiting adenylyl cyclase, hyperpolarizing the neuron, and decreasing serotonin release (Kroeze et al., 2002). 5HT1BRs expressed on non-serotonin neurons can act by similar mechanisms to inhibits release of other neurotransmitters (Heisler et al., 2006). Treatment with a 5HT1BR agonist produced hypophagia and satiety (Halford and Blundell, 1996; Lee and Simansky, 1997). 5HT1BR null mice did not exhibit enhanced adiposity (Bouwknecht et al., 2001). Interestingly, though, genetic or pharmacological inactivation of 5HT1BR blunted responses to d-fenfluramine, suggesting that 5HT1BR and 5HT2CR may act cooperatively to mediate the effects of serotonin on feeding (Lucas et al., 1998; Simansky and Nicklous, 2002; Lee et al., 2004). Studies of feeding patterns indicated that the two receptors may inhibit feeding in somewhat different ways, with 5HT2CR primarily affecting the frequency with which meals are taken and 5HT1BR primarily affecting meal duration (Simansky and Vaidya, 1990; Grignaschi and Samanin, 1992). Heisler and colleagues have established a model of a cooperative relationship between 5HT2CR and 5HT1BR in the arcuate nucleus (Heisler et al., 2006). They found that 5HT1BRs are expressed on AgRP neurons, and that a 5HT1BR agonist produced opposite effects on AgRP and POMC neurons, inhibiting AgRP neurons while exciting POMC neurons. Furthermore, they found that the anorectic effects of 5HT1BR agonists required MC4R. From these data, the authors proposed a model, in which serotonin stimulates the melanocortin system by way of two parallel processes, acting through 5HT2C receptors to directly excite POMC neurons and through 5HT1B receptors to suppress GABA inhibition of POMC neurons by AgRP neurons (Heisler et al., 2006).

While both 5HT2CR and 5HT1BR have anorexigenic effects, other serotonin receptors may promote feeding. Two of these, 5HT1AR and 5HT2BR have recently been reported to exert such effects via mechanisms involving POMC neurons. Early indications of orexigenic function came from studies using 5HT1AR agonists and antagonists. Agonists of 5HT1AR produced hyperphagia while antagonists produced hypophagia (Gilbert et al., 1988; Neill and Cooper, 1988; Moreau et al., 1992). Because 5HT1AR is known to play a prominent role as an inhibitory autoreceptor on serotonergic neurons, these orexigenic effects had been widely attributed to the inhibition of serotonin release. This interpretation has been complicated by a recent study utilizing a POMC-specific knockout of 5HT1AR. This study presented evidence that genetic mutation of 5HT1AR specifically in POMC neurons reduced food intake leading to reduced body weight at 6 months of age (Yadav et al., 2011). This group also reported that a POMC-specific knockout 5HT2BR produced mild hypophagia and a reduction in fat pad mass (Yadav et al., 2009). These effects were attributed to receptors on POMC neurons in the arcuate nucleus. However, POMC is also expressed peripherally, including in cardiomyocytes (Millington et al., 1999). Constitutive mutation of the 5HT2BR resulted in decreased survival and differentiation of cardiomyocytes and was associated with global developmental perturbations (Nebigil et al., 2000). This raises the possibility that the absence of 5HT2BR in peripheral POMC-expressing cells in the heart or elsewhere could contribute to the reduction in body weight reported by Yadav et al.

Like all 5HT2Rs, 5HT2BR is thought to be G_q_-coupled and therefore excitatory, while POMC neurons have a well-established anorexigenic function (Kroeze et al., 2002). The mechanisms through which 5HT2BRs on POMC neurons may produce orexigenic effects have not been established. Reports that 5HT1AR and 5HT2BR on POMC neurons mediate orexigenic effects are especially puzzling since a series of studies recently reported that expression of 5HT2CR on POMC neurons has a critical anorexigenic function (Xu et al., 2008, 2010a). Whether and how multiple types of serotonin receptors might be working at cross-purposes in the same or different population of POMC neurons are open questions which requires additional attention.

## Extrahypothalamic central serotonin

While substantial advances have been made in understanding how serotonin modulates hypothalamic energy balance pathways, there are additional central sites of serotonin action that also warrant consideration. Early indications that serotonin receptors in brainstem nuclei are important for energy balance arose from studies demonstrating that mCPP and d-fenfluramine produced hypophagic effects when injected into the fourth ventricle and that a 5HT2CR antagonist injected into the fourth ventricle blocked hypophagia produced by systemic mCPP injection (Grill et al., 1997; Kaplan et al., 1998). Furthermore, the effects of mCPP and d-fenfluramine were observed even in decerebrate rats, where the direct control of feeding by hypothalamic or other forebrain structures was not possible (Grill et al., 1997; Kaplan et al., 1998).

Within the brainstem, the PBN has particular relevance to energy balance regulation. The PBN receives taste and visceral inputs relayed through the NTS. Interestingly, it also has been shown to receive serotonergic innervation from the dorsal raphe (Petrov et al., 1992). This is unusual, since the vast majority of projections from the dorsal raphe are to the forebrain. Moreover, a substantial number of dorsal raphe neurons were reported to send collateral projections to both the PBN and the PVH in the hypothalamus (Petrov et al., 1992). Neurons of the PBN express both 5HT2CR and 5HT1BR (Bruinvels et al., 1993; Wright et al., 1995). 5HT1BR agonists infused directly into the PBN were found to produce hypophagia, while antagonists attenuated systemic d-fenfluramine-induced hypophagia (Simansky and Nicklous, 2002). Findings such as these indicate that a complete understanding of the serotonergic regulation of energy balance must take into account the functions of brainstem serotonergic circuits.

Another extra-hypothalamic effect of serotonin occurs through the 5HT6 receptor, which is expressed most abundantly in the striatum (Ruat et al., 1993; Ward et al., 1995). Systemic administration 5HT6R antagonists produced hypophagia (Woolley et al., 2001; Perez-Garcia and Meneses, 2005; Heal et al., 2008). Hypophagia also occurred as a result of inactivation of 5HT6R by anti-sense oligonucleotides (Woolley et al., 2001). While genetic nulls of 5HT6R did not exhibit abnormal food consumption on standard chow (Bonasera et al., 2006), they seemed to be resistant to diet-induced obesity when fed a high-fat diet (Frassetto et al., 2008). Taken together, these results indicate that like 5HT1AR and 5HT2BR, 5HT6R signaling produces orexigenic behavior. This again highlights the complexity of serotonergic regulation of energy balance.

## Factors affecting central serotonin synthesis and release

A full understanding of the serotonergic regulation of energy balance requires not only an appreciation of mechanisms through which serotonin-responsive central circuits influence physiological and behavioral determinants of energy balance; it also requires elucidation of the manner in which central serotonergic pathways respond to the organism's nutritional status, circulating nutrients, energy balance hormones, and environmental stimuli.

Serotonin is synthesized from the amino acid tryptophan, which is acquired from the diet, in a two-step process. The rate-limiting step is catalyzed by one of two TPH enzymes, TPH1, which acts exclusively in the periphery, and TPH2, which is expressed primarily in the brain (Fitzpatrick, 1999; Walther et al., 2003; Sakowski et al., 2006). In the CNS, excess serotonin is cleared from the synapse by reuptake into presynaptic terminals via SERT. As serotonin does not cross the blood-brain barrier, central and peripheral serotonin form two distinct pools (Woolley and Shaw, 1954; Merritt et al., 1978). In the brain, serotonin synthesis has been demonstrated to depend on the availability of circulating tryptophan, which is transported across the blood-brain barrier by the L-type amino acid transporter (Fernstrom and Wurtman, 1971; Fernstrom, 2012). Administration of a tryptophan-free diet resulted in a rapid decrease in brain serotonin (Reilly et al., 1997). Conversely, systemic administration of tryptophan increased levels of serotonin and its metabolite 5-HIAA in the brain (Fernstrom and Wurtman, 1971; Schwartz et al., 1990b; Esteban et al., 2004) and also decreased food intake (Morris et al., 1987).

Hypothalamic serotonin is reportedly increased by feeding and decreased by food-restriction (Schwartz et al., 1989, 1990a; Haider and Haleem, 2000). Interestingly though, one study found that when fasted rats were exposed to the sight and smell of food, hypothalamic serotonin concentrations rose to near-maximal levels even before consumption began (Schwartz et al., 1990a) That serotonin levels increased in anticipation of feeding as well as in response to food, indicated that serotonin release in the hypothalamus is not simply a reflection of tryptophan intake during a meal.

A more complex model to explain the relationship between circulating tryptophan and central serotonin has also been proposed. In this scheme, brain serotonin synthesis is dependent not only on levels of circulating tryptophan, but also on levels of other long neutral amino acids (LNAA) that compete with tryptophan for transport into the brain. High tryptophan:LNAA ratios promote central serotonin synthesis, while low tryptophan:LNAA ratios have the opposite effect. Protein-rich meals have been shown to decrease this ratio, while carbohydrate-rich meals increased it, presumably by stimulating insulin release, which in turn promoted absorption of LNAAs by peripheral tissues (Lyons and Truswell, 1988). This mechanism may account for differences in brain serotonin levels measured after consumption of either a high-protein or high-carbohydrate diet (Schweiger et al., 1989). While the utility of such a regulatory mechanism is not clear, one model suggests that hypothalamic serotonin may serve as a feedback sensor to maintain a consistent balance of macronutrients in the diet (Leibowitz and Alexander, 1998).

## Leptin and serotonin

Leptin is an adipocyte hormone known to play a key role in the regulation of energy balance. Circulating leptin levels are well-correlated with levels of adiposity and are believed to provide the CNS with an important indication of body fat stores (Frederich et al., 1995; Considine et al., 1996). Null mutation of the leptin gene (*Ob*) produced significant hyperphagia and severe obesity (Pelleymounter et al., 1995; Chua et al., 1996). Leptin signals through several different receptor isoforms, and in the CNS, the long-form LEPRb predominates (Chua et al., 1996; Elmquist et al., 1998). While leptin does act in the periphery, its actions within the brain are believed to be particularly critical in regulating energy balance (de Luca et al., 2005). LEPRb is expressed in many regions of the brain, and particularly high levels of expression are found in the arcuate, DMH, and VMH (Elmquist et al., 1998), including several populations of neurons that also express serotonin receptors. In the arcuate, LEPRb is expressed in both POMC and AgRP neurons, where they produce the opposite effects, stimulating POMC neurons and inhibiting AgRP neurons (Elias et al., 1999; Cowley et al., 2001). Like serotonin acting through 5HT2CR and 5HT1BR, therefore, leptin is able to stimulate melanocortin signaling by two parallel pathways. Selective elimination of LEPRb on either POMC or AgRP neurons produced a mild obesity phenotype (Balthasar et al., 2004; van de Wall et al., 2008). Interestingly, selective elimination of LEPRb in the steroid factor 1 (SF1)-expressing neurons of the VMH also produced an obesity phenotype, with a cumulative effect in mice lacking LEPRb in both SF1 and POMC neurons, indicating that these may represent distinct parallel pathways (Dhillon et al., 2006; Kim et al., 2011b).

Given their presumably disparate roles in reflecting short-term satiety (serotonin) vs. long-term energy stores (leptin), it has been proposed that serotonin and leptin might represent independent energy-balance systems that integrate in the hypothalamus (Halford and Blundell, 2000). In support of separate but coordinated action of serotonin and leptin, the obesity phenotype of leptin-overexpressing mice on a high-fat diet was exacerbated in the absence of 5HT2CR (Wang and Chehab, 2006). Since 5HT2CR is expressed on both POMC and SF1 neurons (Heisler et al., 2003; Yadav et al., 2009), these represent two potential sites where serotonin and leptin signaling might be integrated. Interestingly, though, a recent study of POMC neurons in the arcuate found that 5HT2CR and LEPRb were expressed on separate populations of POMC neurons, indicating that integration may occur elsewhere (Sohn et al., 2011).

Some recent studies provide evidence that a complex interplay may occur between these two systems. One study examining the impact of combined *Htr2c* null and *ob/ob* leptin null mutations revealed a synergistic effect on glucose regulation, indicated by a marked exacerbation of the diabetes phenotype characteristic of the *ob/ob* genotype (Wade et al., 2008). Early indications that leptin might have a direct effect on serotonin function arose from several histological studies demonstrating that long-form leptin receptor (LEPRb) is expressed in neurons of the raphe nuclei (Elmquist et al., 1998; Mercer et al., 1998; Shioda et al., 1998) where it co-localizes with SERT, a marker of serotonin-producing neurons in the raphe (Collin et al., 2000; Finn et al., 2001). Furthermore, serotonergic neurons of the dorsal raphe have been shown to take up a labeled leptin analog infused into the lateral ventricle, a phenomenon indicative of LEPRb binding and internalization (Fernandez-Galaz et al., 2002). However, a comprehensive analysis of leptin receptor co-localization with serotonin using multiple histochemical techniques including independently generated LepRb reporter mice failed to detect any co-localization with serotonin (Lam et al., 2011).

Evidence for a functional interaction between serotonin and leptin arose from studies demonstrating that central or peripheral leptin administration altered serotonin levels in the brainstem and hypothalamus (Harris et al., 1998; Calapai et al., 1999) Studies by Yamada et al. provided interesting evidence for regulation in the reciprocal direction; treatment with the serotonin precursor 5HTP increased circulating leptin levels (Yamada et al., 1999, 2006). It is unclear whether this effect is mediated by central or peripheral serotonin, as peripheral 5HTP administration affects both. In support of serotonin enhancing leptin levels, SERT null mice, which exhibit globally increased extracellular serotonin levels, have increased serum leptin levels (Chen et al., 2012). Treatment with the TPH2 inhibitor PCPA, which reduces serotonin synthesis and release, increased uptake of leptin in both the hypothalamus and brainstem, providing evidence that serotonin not only regulates leptin release, but also its uptake into the brain (Fernandez-Galaz et al., 2010). Another study by Yamada et al. demonstrated that serotonin may be required for some leptin-mediated influences on energy-balance; PCPA treatment abolished a decrease in refeeding caused by an i.p. leptin injection in fasted mice (Yamada et al., 2003), although this was not replicated in another study which demonstrated that significant serotonin depletion with PCPA had no effect on leptin-induced hypophagia (Lam et al., 2011). This effect was recapitulated by treatment with a 5HT2B/2C receptor antagonist, although not by either 5HT2AR or 5HT1BR antagonists, indicating that either 5HT2BR or 5HT2CR are required for leptin-induced anorexia.

A model in which leptin actions are mediated through serotonergic circuits has recently been proposed by Yadav et al. (2009, 2011). They propose that leptin-mediated suppression of feeding occurs via inhibition of raphe serotonergic neurons. The central experiment of this study utilized a transgenic strategy to eliminate LEPRb specifically in SERT-expressing cells. This manipulation increased food intake, body weight, and body fat and decreased energy expenditure and bone mass (Yadav et al., 2009). Additionally, these mice exhibited reduced hypothalamic expression of anorexigenic melanocortin genes MC4R and POMC and increased expression or orexigenic NPY and AgRP, indicating downregulation of the melanocortin system (Yadav et al., 2009). The degree of perturbation of feeding, body weight, adiposity, metabolism, and gene expression in SERT-specific LEPRb null mice was virtually indistinguishable from *ob/ob* leptin null mice, and POMC- or SF1-specific LEPRb null mice did not display significant alterations in any of these parameters (Yadav et al., 2009). Furthermore, the authors demonstrated that a homozygous (or even a heterozygous) TPH2 null mutation rescued the perturbed feeding and metabolism of leptin null mice (Yadav et al., 2009).

These results conflict with the prevailing notion that leptin exerts its effects on energy balance predominantly through other neuronal populations including POMC neurons of the arcuate (Balthasar et al., 2004; Coppari et al., 2005; van de Wall et al., 2008) and SF1 neurons of the VMH (Dhillon et al., 2006). Consistent with the prevailing notion, a subsequent paper by Lam et al. found that serotonergic neurons in the dorsal raphe neither expressed LEPRb nor were sensitive to leptin, and that depletion of central serotonin with PCPA did not interfere with leptin-induced hypophagia. Furthermore, they reported that, in their hands, SERT-specific LEPRb null mice had no changes in body weight or adiposity (Lam et al., 2011).

In response to concern that the SERT-driver mouse might have produced inactivation of LEPRb outside the serotonergic neurons of the brainstem, a follow-up study by Yadav et al. utilized a tamoxifen-inducible TPH2-driver (Yadav et al., 2011). This allowed inducible inactivation of LEPRb is adult mice, obviating concerns regarding developmental effects in non-serotonergic neurons. A similar phenotype was observed: inducible TPH2-specific LEPRb null mice displayed increased body weight, fat pad weight, food intake, and decreased metabolic rate, dark-cycle locomotor activity, and hypothalamic expression of MC4R and POMC (Yadav et al., 2011). However, in the absence of independent confirmation, these results remain controversial.

## Ghrelin and serotonin

Ghrelin is a gastric hormone that is secreted during periods of fasting, and its levels fall after food intake (Kojima et al., 1999; Date et al., 2000; Tschop et al., 2000). Ghrelin treatment has been shown to increase food intake and chronic administration to result in obesity (Tschop et al., 2000; Wren et al., 2000). Interestingly, there is also evidence that ghrelin is synthesized in a small population of neurons in the arcuate nucleus (Lu et al., 2002; Cowley et al., 2003). The function of hypothalamic ghrelin synthesis is not clear. Peripheral ghrelin crosses the blood-brain barrier, and the arcuate nucleus appears to be an important site for its action. Ghrelin receptors (growth hormone secretagogue receptor, GHSR) are expressed on AgRP neurons, where they mediate excitatory effects (Willesen et al., 1999; Wang et al., 2002). Accordingly, central ghrelin administration activated AgRP neurons and inhibited POMC neurons (Cowley et al., 2003; Riediger et al., 2003). Furthermore, knockout of either MC3R and MC4R or NPY and AgRP produced significant attenuation of the orexigenic effects of peripheral ghrelin (Chen et al., 2004), while ablation of AgRP neurons completely eliminates the effects of ghrelin (Bewick et al., 2005). Ghrelin and serotonin signals appear to produce opposing effects in arcuate nucleus neuronal populations.

Evidence also exists indicating that serotonergic signaling may impact ghrelin release. Both fenfluramine and mCPP decreased plasma active ghrelin levels without altering hypothalamic ghrelin gene expression (Nonogaki et al., 2006). Subsequent work has demonstrated that fenfluramine alters gut motility through a ghrelin-dependent mechanism, inducing motility patterns characteristic of a fed state in fasted rats (Fujitsuka et al., 2009). Furthermore, these effects were shown to be dependent on 5HT2CR but not on MC4R. The authors proposed a model in which 5HT2CR activation decreases gut motility by inhibiting ghrelin release, presumably through descending sympathetic input to the stomach (Fujitsuka et al., 2009). Since serotonin acting in the hypothalamus has been demonstrated to stimulate growth hormone (GH) release from the pituitary (Vijayan et al., 1978; Willoughby et al., 1987), and GH is known to down-regulate production of stomach ghrelin (Nonogaki, 2008), it is also possible that serotonin decreases circulating ghrelin by stimulating GH release. However, another study utilizing hypothalamus explants found that mCPP decreased ghrelin secretion from the hypothalamus itself (Yakabi et al., 2010). There is also evidence that ghrelin can influence serotonin release in both the hypothalamus and hippocampus (Brunetti et al., 2002; Ghersi et al., 2011).

## Insulin and serotonin

Insulin is secreted by the β-cells of the pancreas in response to rising blood glucose levels and it promotes glucose uptake and utilization by peripheral tissues. However insulin also acts in the brain, where it has effects on glucoregulation and energy balance (Woods et al., 1979; Konner et al., 2007; Hill et al., 2010). In the brain, insulin has been shown to act as an anorexigenic hormone: ICV infusion produced hypophagia and weight loss, while a CNS-specific insulin receptor knockout mouse line exhibited obesity (Woods et al., 1979; Bruning et al., 2000). Insulin and serotonin receptors co-localize in several key nuclei of the hypothalamus. Of particular interest, insulin has been demonstrated to act directly on both POMC and AgRP neurons of the arcuate (Konner et al., 2007; Hill et al., 2010). Interestingly, leptin- and insulin-responsive POMC neurons reportedly represent two distinct populations (Williams et al., 2010), and the same has been reported for leptin- and serotonin-responsive neurons (Sohn et al., 2011). It is not yet clear whether 5HT2CR cells are also insulin-responsive. Insulin receptor activation may inhibit the function of 5HT2CR intracellularly in cells expressing both receptors. This has been demonstrated in choroid plexus cells to occur via MAP kinase inactivation of 5HT2CR (Hurley et al., 2003). It is possible that a similar mechanism exists within POMC neurons.

Several lines of evidence now indicate that serotonin and insulin are not merely parallel activators of melanocortin circuits, but that there is an interaction between the two systems. For example, systemic administration of 5HT2CR agonists decreased serum insulin at doses that did not impact food intake or body weight (Zhou et al., 2007). This effect was demonstrated to occur via an MC4R dependent-mechanism. Additionally, Orosco et al. reported that an infusion of fenfluramine directly into the hypothalamus increased hypothalamic extracellular insulin levels (Orosco et al., 2000). In support of a more general effect, systemic dexfenfluramine treatment increased serum insulin levels (Papazoglou et al., 2012). SERT-deficient mice have increased serum insulin as well as increased pancreatic islet cell density and other morphological changes indicative of increased insulin production (Chen et al., 2012).

## Orexin and serotonin

Orexin neurons are located in the lateral hypothalamus and project widely throughout the brain. Serotonergic neurons of the dorsal raphe nucleus express orexin receptors (Marcus et al., 2001; Wang et al., 2005). Serotonergic raphe neurons receive a particularly dense innervation from orexin neurons (Peyron et al., 1998; Nambu et al., 1999; Brown et al., 2002). Moreover, serotonergic neurons were found to be stimulated by orexin administration in a raphe slice preparation (Brown et al., 2002; Liu et al., 2002). However, this effect may not have occurred solely through direct activation of orexin receptors on serotonin neurons. Another study has shown that orexin inhibited excitatory glutamatergic input to dorsal raphe serotonin neurons via a retrograde endocannabinoid signal (Haj-Dahmane and Shen, 2005). Behavioral effects produced by orexin administration were blocked by 5HT2AR and 5HT2CR antagonists (Duxon et al., 2001; Matsuzaki et al., 2002), raising the possibility that serotonergic systems contribute to the behavioral actions of orexin.

There is also evidence that serotonin systems can directly impact the function of orexin neurons. Orexin neurons in the lateral hypothalamus express 5HT1A receptors and serotonergic nerve terminals are in close apposition to orexin neurons (Muraki et al., 2004). Additionally, administration of serotonin and 5HT1AR agonists inhibited orexin neurons (Muraki et al., 2004). Functionally, mice lacking orexin neurons did not exhibit 5HT1AR antagonist-induced hyperlocomotion (Muraki et al., 2004). Another study found that impaired expression of both orexin and POMC by RNA interference abolished the hypophagia produced by mCPP treatment, while reduced expression of either gene alone had no effect (Nonogaki and Kaji, 2010). Taken together, these results indicate that orexin neurons may play a role in mediating the anorexigenic effects of 5HT2CR or 5HT1BR stimulation, as well as the hyperlocomotor effects of 5HT1AR activation.

## Peripheral serotonin

In mammals, the vast majority of serotonin resides not in the brain, but in the periphery (Erspamer, 1966). In contrast to the CNS, where serotonin is synthesized only by a small number of neurons in a few discrete nuclei, peripheral serotonin is produced by both neurons and non-neuronal cells in a wide variety of tissues. However, by far the largest quantity of serotonin resides within the gut, in specialized endocrine cells, called enterocromaffin cells that synthesize and store large quantities of serotonin (Erspamer, 1966). Serotonin release by these cells is stimulated by the presence of food and in turn, serotonin stimulates gut motility (Racke et al., 1996; Fujimiya et al., 1997; Kidd et al., 2008). This circuit is therefore critical for proper absorption of nutrients.

Enterochromaffin cells are found in the enteric epithelium throughout the length of the digestive track (Erspamer, 1966). These cells release moderate levels of serotonin constitutively and large quantities in response to various relevant factors including decreased pH and mechanical pressure (Racke et al., 1996; Fujimiya et al., 1997). Serotonin is released primarily into the underlying connective tissue layer of the mucosa, which is innervated by nerve terminals of the primary sensory neurons of the enteric nervous system. From there, high concentrations of serotonin spill over into both the circulatory system and the intestinal lumen (Gronstad et al., 1985; Nilsson et al., 1987; Fujimiya et al., 1997; Bearcroft et al., 1998). Furthermore, it has been demonstrated that SERT is widely expressed on enteric epithelial cells, allowing them to clear excess serotonin, preventing 5HTR desensitization (Wade et al., 1996; Chen et al., 1998). Mice lacking functional SERT have excessive gut motility and gastrointestinal dysfunction (Chen et al., 2001).

The primary sensory neurons of the enteric submucosa are cholinergic and their function is required for the proper peristaltic and secretory responses to food (Cooke et al., 1997; Pan and Gershon, 2000). Their dendritic terminals extend into the mucosa and respond to serotonin via an as yet unidentified receptor, known as 5HT1P (Branchek et al., 1988; Wade et al., 1991). These neurons synapse onto cholinergic interneurons within the myenteric plexus, which in turn synapse onto excitatory and inhibitory motor neurons, causing rhythmic peristalsis (Grider et al., 1996). Presynaptic 5HT4R receptors at both of these synapses enhance neurotransmitter release (Pan and Galligan, 1994; Galligan et al., 2003). Stimulation of 5HT1P seems to be important in initiating peristalsis in response to a food bolus, while 5HT4R is involved in maintaining peristalsis once it has begun (Grider et al., 1996; Gershon, 2004, 2005). There is also evidence for serotonin causing smooth muscle contraction in the stomach fundus via 5HT2BR located directly on smooth muscle cells (Kursar et al., 1994; Depoortere et al., 2006).

5HT3 receptors are expressed on nerve terminals of vagal afferents located in the gastrointestinal track, where they respond to serotonin released by enterochromaffin cells (Glatzle et al., 2002; Raybould et al., 2003). These afferents mediate sensations of gastric discomfort and nausea, and 5HT3R antagonists are effective anti-emetic drugs (Gershon, 2004, 2005). Some of these vagal afferents make connections with regions of the brain important to energy balance. Serotonin release resulting from gastric distension led to increased neuronal activity in regions including the NTS and PVH (Mazda et al., 2004). This effect was blocked by either truncal vagotomy or peripheral (but not central) administration of a 5HT3R antagonist (Mazda et al., 2004).

Some serotonin released by enterochromaffin cells is absorbed into the circulation by capillaries in the enteric submucosa, where it enters the blood stream (Tamir et al., 1985; Bearcroft et al., 1998). This serves as the primary source of circulating serotonin (Bertaccini, 1960) and levels of plasma serotonin increase after a meal (Bearcroft et al., 1998; Houghton et al., 2003). In the blood, serotonin is taken up by platelets, which express SERT (Tamir et al., 1985; Ni and Watts, 2006). Serotonin stored in platelets serves as an important reservoir for peripheral serotonin. The inability of serotonin to cross the blood-brain barrier limits the ability of circulating serotonin fluctuation to impact energy balance circuits in the CNS (Woolley and Shaw, 1954; Merritt et al., 1978). However, circulating serotonin acting in the periphery has many important functions, some of which relate to energy balance. Like central infusion of serotonin, peripheral administration of serotonin decreased food consumption and accelerated satiety (Fletcher and Burton, 1986; Edwards and Stevens, 1991).

Circulating serotonin has also been shown to have complex effects on peripheral glucose regulation. Peripheral serotonin seems to impact glucoregulation via at least two discrete apparently opposing mechanisms. Peripheral serotonin administration has been found in some studies to increase, and in others to decrease circulating blood glucose levels, with the discrepancy possibly depending on dose, route, or other conditions (Yamada et al., 1989, 1995; Sugimoto et al., 1990). Serotonin-induced hyperglycemia is likely due to inhibition of glucose uptake by the liver and muscle tissue (Hajduch et al., 1999; Moore et al., 2005). Conversely, peripheral serotonin also produced hyperinsulinemia, an action promoting reduction of glucose levels. This effect presumably occurs via stimulation of pancreatic β-cells by serotonin. β-cells actually synthesize and store serotonin (Ekholm et al., 1971) and recent evidence indicated that an interesting mechanism may at least partially underlie regulation of insulin release by serotonin. Serotonin in β-cells has been reported to act intracellularly via a 5HTR-independent mechanism, whereby serotonin directly binds to and activates small GTPase molecules to stimulate insulin secretion (Paulmann et al., 2009).

In addition, circulating serotonin influences lipid metabolism. Peripheral administration of serotonin accelerated lipid metabolism, decreasing circulating levels of triglycerides, fatty acids, and cholesterol (Watanabe et al., 2010). This has been attributed to increased release and turnover of bile acid (Bogach and Liashchenko, 1976; Watanabe et al., 2010). Functional relevance is lent to this finding by another recent study showing that blood serotonin was substantially elevated in diet-induced obese mice (Kim et al., 2011a).

## Conclusion

In conclusion, the extent to which central and peripheral serotonin signaling pervasively impact the regulation of energy balance is quite remarkable. It is challenging to identify central or peripheral neural mechanisms of energy balance regulation that are NOT sensitive to serotonergic modulation. The use of mouse genetic models with cell-type-specific patterns of gene inactivation is contributing substantially to rapid progress in this field. The most extensively characterized of the serotonergic influences on energy balance pathways relates to the modulation of arcuate nucleus POMC and NPY/AgRP neuronal populations. Clearly, this has significant functional relevance. However, the serotonergic innervation of additional hypothalamic regions and the expression of multiple 5HTR subtypes in these regions suggest that much remains to be learned regarding serotonergic regulation of hypothalamic energy balance pathways other than those originating the arcuate nucleus. Also important, but less understood, are mechanisms through which serotonin systems regulate energy balance pathways in caudal brainstem regions such as the NTS and PBN. In addition to advances in understanding how serotonergic inputs influence energy balance pathways, new light is being shed on mechanisms through which energy balance hormones influence the activity of serotonin systems. It is also becoming clear that serotonergic influences on hypothalamic energy balance pathways interact with those mediated by leptin, ghrelin, and insulin. Evidence that serotonin systems can influence circulating levels of these hormones indicates an additional level of complexity with regard to the interplay of these signaling molecules in energy balance regulation. Finally, newly uncovered roles for central and peripheral serotonin in the regulation of glucose homeostasis and lipid metabolism underscore the pervasive involvement of serotonin signaling in the interplay between central and peripheral mechanisms of energy balance regulation.

### Conflict of interest statement

The authors declare that the research was conducted in the absence of any commercial or financial relationships that could be construed as a potential conflict of interest.
